# Supplementary material for: Content-rich biological network constructed by mining PubMed abstracts
Source: BMC Bioinformatics. 2004 Oct 8;5:147. doi: 10.1186/1471-2105-5-147 (PMC528731; doi:10.1186/1471-2105-5-147)
Supplement: Additional File 2 — The original results of the above study (non-essential files are deleted to keep the file size under the limit set by BMC bioinformatics). [file 1471-2105-5-147-S2.bz2 › chilibotAdditionalFile2/dip10/337ID8621492E4139/html/LTA_LTB.html]

 


 **LTA** and **LTB** 
  
Found 137 abstracts in PubMed, retrieved 10.  
 

 What does Google say? 
 PDF only 
| .edu only 

---

**Interactive relationship** (e.g. stimulation, inhibition, etc)

**Neutral relationship**- LT consists of soluble LT alpha  [ **LTA** ]  3, which binds to TNF R1 and TNF R2, and membrane LT alpha  [ **LTA** ]  1 beta 2, which binds to LT beta  [ **LTB** ]  R.  Ref: 12882833 Int Immunol, 2003

**Non-interactive relationship** (e.g. studied together, co-existance, homology, etc.)

- Lymphotoxin alpha  [ **LTA** ] , lymphotoxin beta  [ **LTB** ] , C C chemokine ligand CCL 21, CXC chemokine ligand CXCL 12, CXCL13, and CCL22 were increased in thyroids from autoimmune patients.  Ref: 12794165 J Immunol< MedlineTA>J Immunol, 2003
- Expression of cytokines TNF beta  [ **LTA** ]  and LT beta  [ **LTB** ]  preceded, and the expression of TGF beta1 followed chemokine upregulation.  Ref: 12828562 Scand J Immunol, 2003
- This activity of LT depends on membrane LT alpha  [ **LTA** ]  1 beta 2 and not soluble LT alpha  [ **LTA** ]  3, since DA virus persisted and induced inflammatory demyelination in LT beta  [ **LTB** ]  R, but not TNF R1 or TNF R2, mice.  Ref: 12882833 Int Immunol, 2003
- Functional studies in gene knockout and transgenic mice systems have shown that lymphotoxin alpha  [ **LTA** ]  and lymphotoxin beta  [ **LTB** ]  LT alpha  [ **LTA** ]  and LT beta  [ **LTB** ]  are of fundamental importance in peripheral lymphoid organ development, but it remains unclear what role these cytokines have to play in the adult immune response and in the pathogenesis of disease.  Ref: 12474234 J Pathol, 2003
- The LT alpha  [ **LTA** ]  and LT beta  [ **LTB** ]  R mice failed to mount a virus specific cytotoxic T cell response.  Ref: 12882833 Int Immunol, 2003
- ... the  **LTA**  4 hydrolase inhibitor SC57461A and the LT biosynthesis inhibitor MK 0591 inhibited AA but not  **LTB**  4 mediated PLD activation.  Ref: 12660228 J Leukoc Biol, 2003
- Neither lymphotoxin alpha  [ **LTA** ]  nor lymphotoxin beta  [ **LTB** ]  receptor expression is required for biogenesis of lymphoid aggregates or differentiation of natural killer cells in the pregnant mouse uterus.  Ref: 12603600 Immunology, 2003
- There was relatively little demyelination associated with inflammation in LT alpha  [ **LTA** ]  and LT beta  [ **LTB** ]  R mice compared to susceptible SJL mice, suggesting the possibility that LT plays a role in mediating demyelination.  Ref: 12882833 Int Immunol, 2003
- Samples homozygous at TNFA 308 and BAT1 intron 10 demonstrated identity between the African TNFA 308\*2 haplotype, the 8.1AH and the Asian diabetogenic 58.1AH HLA A33, B58, DR3 across a region spanning BAT1, ATP6G, IKBL,  **LTA** , TNFA,  **LTB** , LST 1 and AIF 1.  Ref: 12859597 Tissue Antigens, 2003
- To investigate LT alpha  [ **LTA** ]  beta s activities in lymphoid organogenesis, mice simultaneously expressing LT alpha  [ **LTA** ]  and LT beta  [ **LTB** ]  under rat insulin promoter II RIP control were compared with RIPLT alpha mice in a model of lymphoid neogenesis and with LT beta  [ **LTB** ]  mice.  Ref: 12732657 J Exp Med, 2003
